# Does Telehealth Monitoring Identify Exacerbations of Chronic Obstructive Pulmonary Disease and Reduce Hospitalisations? An Analysis of System Data

**DOI:** 10.2196/medinform.6359

**Published:** 2017-03-22

**Authors:** Melissa Kargiannakis, Deborah A Fitzsimmons, Claire L Bentley, Gail A Mountain

**Affiliations:** ^1^ Faculty of Health Sciences Western University London, ON Canada; ^2^ School of Nursing and Allied Health Liverpool John Moores University Liverpool United Kingdom; ^3^ School of Health and Related Research University of Sheffield Sheffield United Kingdom; ^4^ Faculty of Health Studies University of Bradford Bradford United Kingdom

**Keywords:** information systems, telemedicine, pulmonary disease, chronic obstructive, triggers and rules, information integration, decision support systems, information retrieval

## Abstract

**Background:**

The increasing prevalence and associated cost of treating chronic obstructive pulmonary disease (COPD) is unsustainable. Health care organizations are focusing on ways to support self-management and prevent hospital admissions, including telehealth-monitoring services capturing physiological and health status data. This paper reports on data captured during a pilot randomized controlled trial of telehealth-supported care within a community-based service for patients discharged from hospital following an exacerbation of their COPD.

**Objective:**

The aim was to undertake the first analysis of system data to determine whether telehealth monitoring can identify an exacerbation of COPD, providing clinicians with an opportunity to intervene with timely treatment and prevent hospital readmission.

**Methods:**

A total of 23 participants received a telehealth-supported intervention. This paper reports on the analysis of data from a telehealth monitoring system that captured data from two sources: (1) data uploaded both manually and using Bluetooth peripheral devices by the 23 participants and (2) clinical records entered as nursing notes by the clinicians. Rules embedded in the telehealth monitoring system triggered system alerts to be reviewed by remote clinicians who determined whether clinical intervention was required. We also analyzed data on the frequency and length (bed days) of hospital admissions, frequency of hospital Accident and Emergency visits that did not lead to hospital admission, and frequency and type of community health care service contacts—other than the COPD discharge service—for all participants for the duration of the intervention and 6 months postintervention.

**Results:**

Patients generated 512 alerts, 451 of which occurred during the first 42 days that all participants used the equipment. Patients generated fewer alerts over time with typically seven alerts per day within the first 10 days and four alerts per day thereafter. They also had three times more days without alerts than with alerts. Alerts were most commonly triggered by reports of being more tired, having difficulty with self-care, and blood pressure being out of range. During the 8-week intervention, and for 6-month follow-up, eight of the 23 patients were hospitalized. Hospital readmission rates (2/23, 9%) in the first 28 days of service were lower than the 20% UK norm.

**Conclusions:**

It seems that the clinical team can identify exacerbations based on both an increase in alerts and the types of system-generated alerts as evidenced by their efforts to provided treatment interventions. There was some indication that telehealth monitoring potentially delayed hospitalizations until after patients had been discharged from the service. We suggest that telehealth-supported care can fulfill an important role in enabling patients with COPD to better manage their condition and remain out of hospital, but adequate resourcing and timely response to alerts is a critical factor in supporting patients to remain at home.

**Trial Registration:**

International Standard Randomized Controlled Trial Number (ISRCTN): 68856013; http://www.isrctn.com/ISRCTN68856013 (Archived by WebCite at http://www.webcitation.org/6ofApNB2e)

## Introduction

Chronic obstructive pulmonary disease (COPD) is the fifth-highest cause of mortality and second-highest cause of emergency admissions to hospital in the United Kingdom [1]. It costs the National Health Service (NHS) more than £800 million per annum [2]. For hospital patients, COPD accounts for £587 million of the total £1.08 billion spent on admissions for lung disease by the NHS [3,4]. Patients discharged from hospital following COPD exacerbations have a high readmission rate [5]. The forecasted increase in COPD prevalence makes current models of care delivery unsustainable. There is a global need for care delivery models that encourage prevention, self-management [6], and home-based management approaches designed to avoid hospital admission and reduce health care costs [7].

### Definitions

Chronic obstructive pulmonary disease is characterized by progressive worsening of lung capacity. Patients with advanced COPD typically experience impaired physical, emotional, and social functioning, which results in poor quality of life [8]. The NHS describes COPD as progressive airflow obstruction that is not fully reversible and does not change markedly over several months [9].

Exacerbations of COPD are described as “a sustained worsening of the patient’s symptoms from their usual stable state, which is beyond normal day-to-day variations, and is acute in onset” [8]. Key symptoms indicative of an exacerbation include increased dyspnea; sputum purulence; sputum volume; cough, wheeze, or fatigue; chest tightness; reduced exercise tolerance; fluid retention; or acute confusion [9-14]. Segrelles et al [15] identify one addendum “...that leads to a change in medication” and note that patients with more acute exacerbations of COPD (AECOPDs) have a worse prognosis. Toy et al [16] have identified that patients with COPD are likely to experience exacerbations that are unreported. The severity of AECOPD is closely related to health care delivery costs [17]. Fernández-Granero et al [11] were able to detect AECOPDs an average of 4.8 days before onset with 80.5% accuracy using a questionnaire analyzed by a probabilistic neural network, but this approach is not part of the standard care pathway and adds an incremental step. If telehealth monitoring embedded within a clinical support service is able to provide early and accurate detection of AECOPDs, as suggested by Fernández-Granero et al’s results [11], it could offer an opportunity for early intervention to alleviate symptoms and reduce care costs.

### Local Context

The region chosen for this pilot study has a high prevalence of COPD linked to the predominant mining industry [16]. The Index of Multiple Deprivation rates this region as one of the most deprived due to poor diet and other adverse lifestyle factors, including a relatively high level of smoking [18]. Between April 2006 and March 2007, COPD-related admissions billed to the local health service cost £2.2 million [14].

### Telehealth Intervention

The telehealth-monitoring intervention was introduced with the goals of decreasing hospitalizations, improving the quality of life for patients, and reducing resource use, while significantly increasing capacity of the service. It was believed that the data collected through the telehealth system would enable clinicians to provide a more patient-centered service by identifying whether patients required additional supportive home visits to address any fluctuations in their condition. Using these data, it was also hoped that unnecessary visits could be eliminated, thereby freeing resources that could be used to support additional patients.

The selected telehealth system (Doc@Home) provided both monitoring and self-management support functionality. Using a small hand-held device, patients were required to answer tailored questions about their health status by reading questions on the screen of the device and pressing the appropriate response button. Patients also used a blood pressure monitor and oximeter peripherals to measure their blood oxygen levels each day. The peripherals were connected by Bluetooth to the hand-held device, and all readings were transmitted to a secure Web-based server by telephone line, ready for access by the clinicians. Patients were able to observe their readings each day; this was a core educational element of the service. If reported signs and symptoms fell outside clinician-generated thresholds, or if the patient failed to undertake the monitoring activity, the system generated a color-coded alert visible to the remote clinician when reviewing the data submitted by patients for that day. Further details of the intervention are available online [19].

Installation of the telehealth equipment involved the installer instructing patients (and also their carer, if appropriate) on how to use the equipment, including the peripherals. The installer also informed them of when they should take readings as well as how and when they might request help if required. The installer provided the patient with a customized instruction manual, which included service information and key contact details should they require assistance.

### Outcome Measures

If telehealth monitoring can identify AECOPDs, it could provide an opportunity for clinicians to intervene and prevent more invasive and more costly interactions, such as hospital admissions. Consequently, the primary outcome measure of interest was the proportion of participants readmitted to hospital with COPD during the 8-week intervention and 6-month follow-up, determined using patient-level data on hospital readmissions obtained from the Secondary Uses Service (SUS), the single, comprehensive repository for health care data in England [19]. The secondary outcome measure of interest was the proportion of patients requiring unscheduled health care support for the 8-week intervention period and 6-month follow-up, determined through analysis of SUS data [19].

The aim of this paper is to show whether telehealth monitoring can identify AECOPDs for patients with COPD that require follow-up and, potentially, a clinical intervention.

## Methods

This paper reports on the analysis of data captured by the telehealth monitoring system during a pilot study [20], completed in preparation for a randomized controlled trial (RCT) of telehealth monitoring for patients with early-stage COPD [19]. The pilot study was conducted over a period of 14 months. Full details of the intervention and the study are available in the published initial findings [20].

### Recruitment

Full details of the eligibility criteria, recruitment, and consent procedures used for the pilot study are detailed in the initial published findings of the study [19]. A total of 23 participants were recruited to the experimental group receiving the telehealth-supported intervention.

### Data Collection

The telehealth monitoring system captured data from two sources: (1) data uploaded by the 23 participants providing telehealth-monitoring data as part of an 8-week early supported discharge nursing intervention and (2) clinical records entered as nursing notes by the clinicians.

We also requested SUS data from the local Primary Care Trust Commissioner on the frequency and length (bed days) of hospital admissions, frequency of hospital Accident and Emergency (A&E) visits that did not lead to hospital admission, and frequency and type of community health care service contacts other than the COPD discharge service for all participants (who completed the 8-week intervention) for the duration of the intervention and 6-month follow-up.

### Statistical Analysis

The patient input data used by the clinicians to determine whether patients may be experiencing an AECOPD were collected as nominal dichotomous data with a “yes/no” alert-triggering system: the patient’s inputs did or did not trigger an alert. We calculated the frequency with which each patient generated an alert for each prompt each day, if any.

Hospital admission data were tracked for all 23 participants for 6 months following the end of the intervention. For each patient, we identified the system-generated alerts, any resultant clinical activity, and the need for additional supportive services including visits to a hospital A&E or hospital admissions.

### Ethics and Governance

The pilot study received ethical approval from the South Yorkshire Research Ethics Committee (reference 10/H130/48) and research and development approval from the local NHS hospital Trust in the United Kingdom. Approval was subsequently obtained from Western University in London, ON, Canada, for the analysis of the quantitative telehealth system data.

## Results

This paper reports on the 23 participants undertaking an 8-week early supported discharge nursing intervention. If the patient was still considered to be too unwell to be discharged from the service at the end of the 8 weeks, the clinicians allowed the participant to retain the equipment until they were admitted into a community nursing program to provide additional support to patients with advanced COPD. Removal of the telehealth monitoring system and discharge of the patients from the service was subject to the availability of the patient, a member of the clinical team, and a technician. Consequently, the number of potential data entry days varied for each patient. We have reported on the 42 days after system installation that all patients provided data (referred to as period 1) and the additional data captured by the telehealth monitoring system after day 42 until removal of the equipment (referred to as period 2).

### Use of the Telehealth Monitoring System

The entire cohort provided data for a minimum of 42 days following installation of the equipment, with a mean duration of data provision of 51.1 (SD 7.4) days. As shown in Table 1, two patients were subject to delayed discharge from the service, undertaking telehealth monitoring for an additional 2 weeks. Data were provided by patients for 92.43% (1086/1175) of the time the system was installed, and any explanations for missing data are included in Table 1.

**Table 1:** Total alerts generated by patients with identification of clinical interventions.

Patient ID^a^	Total alerts generated (n=512), n	Days on the telehealth system (n=1175), n	Missed data input (n=89)		Days ≥1 alert (n=243), n (%)	Days without an alert (n=932), n (%)
			Days, n	Reason			
P4^b,c^	88	48	10	In hospital	25 (52.1)	23 (47.9)
P23^b,c^	84	51	0		32 (62.7)	19 (37.3)
P3^b,c^	72	56	10	In hospital	26 (46.4)	30 (53.6)
P13^b,c^	36	69	15	10 in hospital	13 (18.8)	56 (81.2)
P16	32	47	0		22 (46.8)	25 (53.2)
P19^b,c^	29	54	3	Faulty system	13 (24.1)	41 (75.9)
P2^b^	23	71	7	5 on holiday	15 (21.1)	56 (78.9)
P14^b^	21	59	9		13 (22.0)	46 (78.0)
P10^b^	16	50	0		12 (24.0)	38 (76.0)
P11^b^	13	42	5	4 in hospital	6 (14.3)	36 (85.7)
P6^b^	13	47	0		13 (27.7)	34 (72.3)
P15^b,c^	12	47	0		7 (14.9)	40 (85.1)
P5	12	46	0		6 (13.0)	40 (87.0)
P17	11	52	0		6 (11.5)	46 (88.5)
P9^b,c^	11	49	0		7 (14.3)	42 (85.7)
P21^b,c^	10	49	5		5 (10.2)	44 (89.8)
P22^b^	9	52	1	Forgot	9 (17.3)	43 (82.7)
P7	6	42	0		5 (11.9)	37 (88.1)
P20	6	45	4		2 (4.4)	43 (95.6)
P1	5	51	12		4 (7.8)	47 (92.2)
P12	2	43	0		1 (2.3)	42 (97.7)
P18	1	50	8	Holiday	1 (2.0)	49 (98.0)
P8	0	55	0		0 (0.0)	55 (100.0)

^a^ Patient numbers are anonymized.

^b^ Patients who received treatment intervention.

^c^ Patient who were hospitalized.

### Volume and Frequency of Alerts

The 12 telehealth monitoring system prompts and four Bluetooth readings provided by participants—with responses that triggered a system alert—were mapped to the NHS’ AECOPD key symptoms and are shown in Table 2.

**Table 2:** NHS AECOPD symptoms paired with Doc@Home prompts.

Doc@Home prompts		Response-triggering alert	Related NHS AECOPD key symptoms
1. Have you been feeling more tired than usual over the last 24 hours?		More than usual	Increased fatigue / reduced exercise tolerance
2. How is your breathlessness today?		More than usual	Increased dyspnea, wheeze, and/or cough
	2a. Is that on normal activities/ exertion or rest?	Doing less than usual	Increased dyspnea, wheeze, and/or cough
3. Has your sleep been affected by coughing or shortness of breath?		More than usual	Increased dyspnea, wheeze, and/or cough
4. Have you produced sputum in the last 24 hours?		More than usual	Increased sputum purulence and increased sputum volume
	4a. What color is your sputum?	Any if quantity is more than usual	Increased sputum purulence and increased sputum volume
5. Are your ankles or feet swollen this morning?		More than usual	Fluid retention
6. How able are you to do your self-care activities (dressing/ bathing)?		Not at all	Increased fatigue / reduced exercise tolerance
7. Have you had to use your relieving medication in the last 24 hours?		More than usual	None
8. How anxious have you been over the last 24 hours?		Much more than usual	None
	8a. How have you coped with your anxiety?	Any if anxiety level much more than usual	None
9. How has your general health been in the last 24 hours? (1=normal for me; 10=extremely poor)		No response captured in the system	None
10. Have you had any problems in walking about in the past week? (yes/some/no)		No response set to trigger alert	Increased fatigue / reduced exercise tolerance
11. Have you had problems doing your usual activities in the past week?		No response set to trigger alert	Increased fatigue / reduced exercise tolerance
12. Have you had to contact the following within the last 24 hours? (no-one, COPD service, GP, hospital)		No response set to trigger alert	None
13. Blood pressure-Bluetooth systolic blood pressure (mmHg)		Personally adjusted parameter	None
14. Blood pressure-Bluetooth diastolic blood pressure (mmHg)		Personally adjusted parameter	None
15. SpO_2_ reading-Bluetooth O2 (%)		Personally adjusted parameter	None
16. SpO_2_ reading-Bluetooth pulse rate (bpm)		Personally adjusted parameter	None

**Figure 1:**
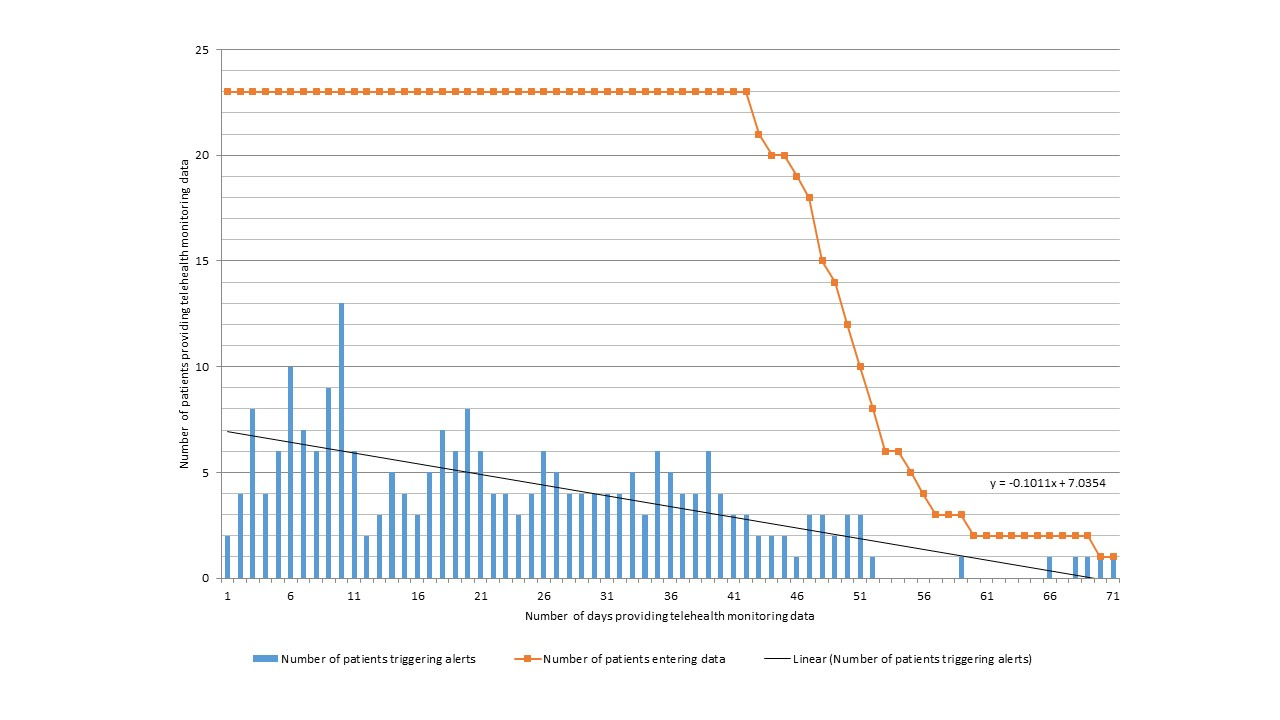
Number of patients entering data and number of patients triggering alerts by day of using the equipment (N=23).

As shown in Figure 1, most patient alerts were triggered during the first 10 days following installation of the telehealth-monitoring equipment. The mean number of patients triggering alerts each day declined over time, with a slope of equation y=–0.1011x + 7.0354. A mean of 30% (6.9/23) of the patient cohort generated alerts each day during the first 10 days following installation of the equipment. This reduced to a mean of 20% (4.5/23) from days 11 to 42, and decreased further to a mean of 14% (1.0/7.2) of the patient cohort triggering alerts each day for days 43 to 71.

As depicted in Table 1, patients experienced, on average, four times as many days without generating alerts (932/1175, 79.32%) than with generating alerts (243/1175, 20.68%).

**Figure 2:**
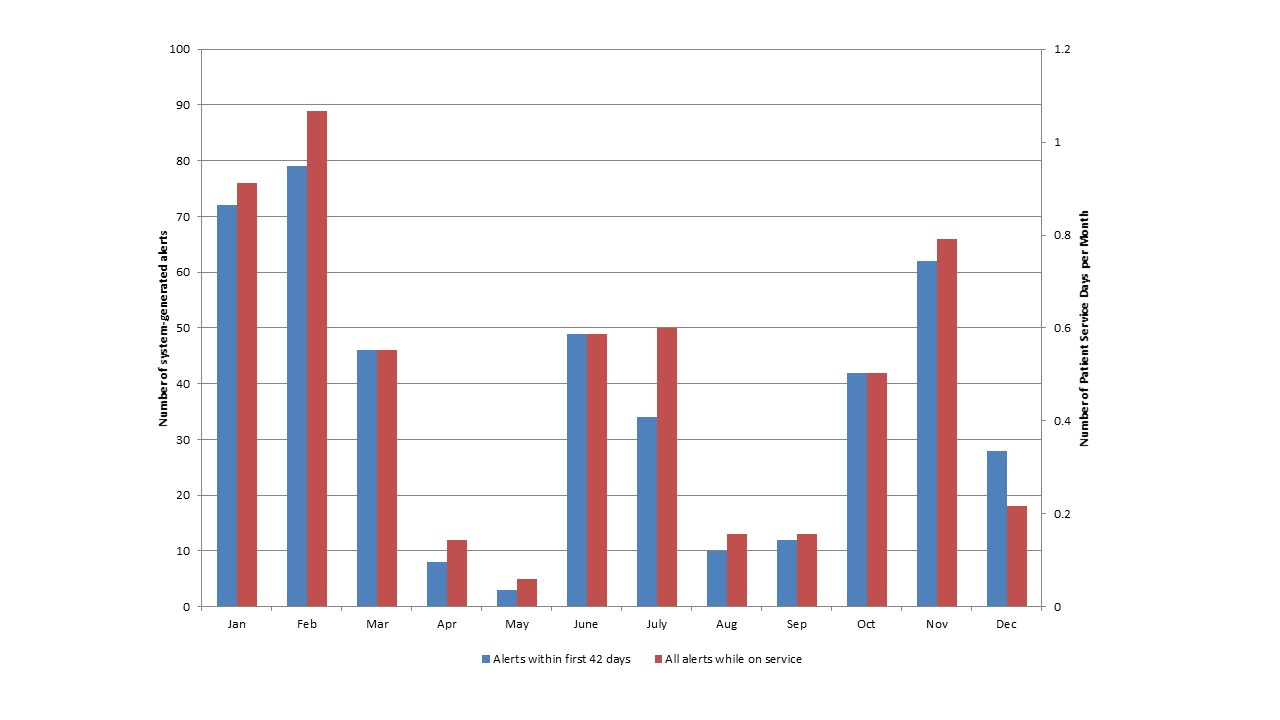
Number of system-generated alerts per month.

**Figure 3:**
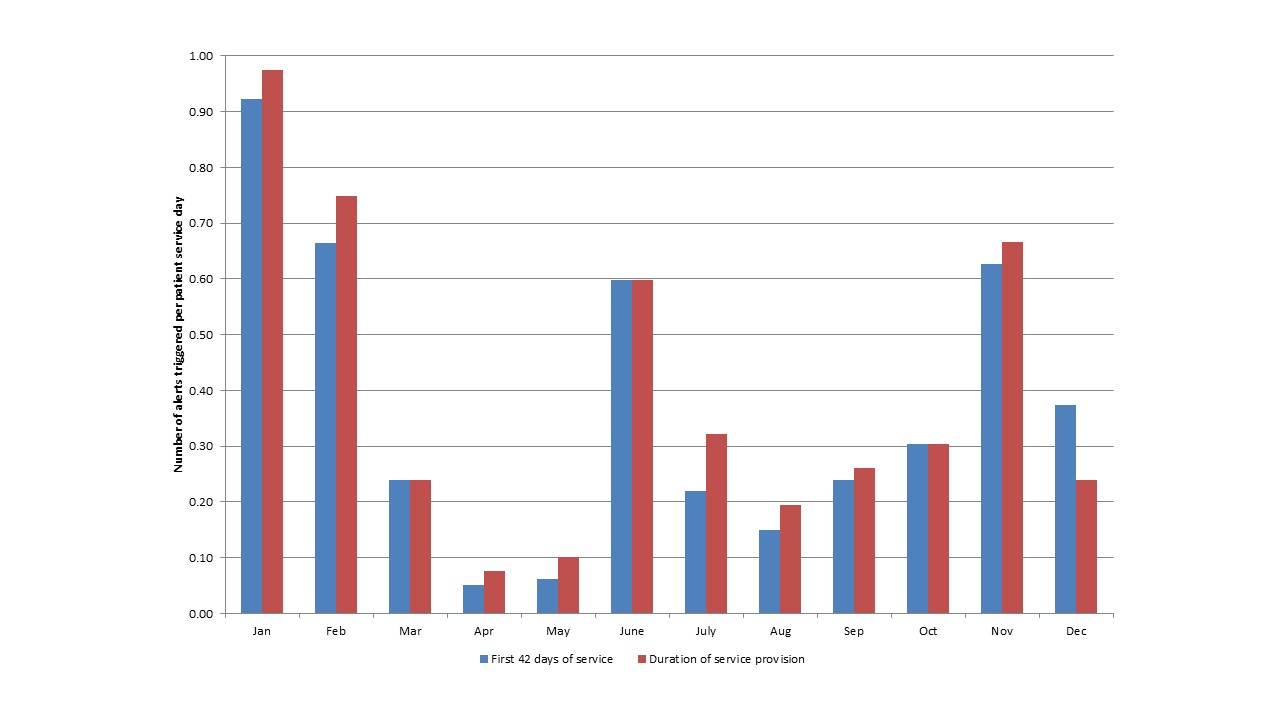
Number of system-generated alerts triggered per patient service day.

Figure 2 suggests that there is a seasonal effect to the system-generated alerts. When controlling for the differing number of patients receiving the service each month, Figure 3 shows that there was a significant decrease in the number of alerts triggered per patient service day during the spring months of March to May. The number of system-generated alerts per patient service day in December were much lower than for the months of November and January.

### Alert Triggers

For the 512 alerts triggered during period 1 and period 2, almost half were triggered by just two metrics: feeling more tired than usual (23.4%, 120/512) and taking relieving medication more frequently (23.4%, 120/512), as shown in Table 3. Additional self-reporting (changes in sputum volume or color, increased breathlessness, increased anxiety, disturbed sleep caused by coughing or breathlessness, and swollen feet) accounted for 19.3% (99/512) of triggers. Changes in physiological conditions (blood pressure, oxygen levels, and heart rate) accounted for the remaining 25.8% of alerts (132/512).

**Table 3:** Patient alerts grouped by trigger (n=512).

Trigger	Patient alerts, n (%)
Feeling more tired than usual	120 (23.4)
Taking relieving medication more often	120 (23.4)
Blood pressure (systolic and/or diastolic)	74 (14.5)
SpO_2_ (%) and heart rate	58 (11.3)
Sputum (color or volume)	50 (9.8)
Difficulty with self-care	41 (8.0)
Increased breathlessness	18 (3.5)
Increased anxiety	14 (2.7)
Sleep disturbed by coughing/shortness of breath	9 (1.8)
Swollen feet	8 (1.6)

### Clinical Response to Alerts

The nursing notes identified 360 interventions that were undertaken during the study. Clinician interventions undertaken during the study, including responses to system-generated alerts, are identified in Table 4.

**Table 4:** Alert subcategories, frequency, definitions, and examples of interventions (n=360).

Interventions	Total	Definition/inclusion criteria	Examples
Patient metric review	104	Nursing notes indicated patient within appropriate parameters, or input data “satisfactory”/“stable” so no intervention required	“Trends/Parameters reviewed and all appear satisfactory” [Nurse2]
Patient contact	81	Telephone contact with patient	“Patient contacted by phone regarding his blood pressure, he states that as soon as he is required to check his BP he becomes anxious. In view of this advised him to refrain from checking this and that we will visit next week and check it manually. He is well otherwise and does not require a visit sooner” [Nurse 3]
Orange^a^	52	Nursing notes comment alert changed to orange	“Alert generated [date] passed to Nurse X [date]. Alert changed to orange” [Nurse 7]
Treatment	39	Clinicians provided lifestyle recommendations, medication instructions, or referred patients to seek medical attention from other “clinics”	“Telephoned patient who stated he was more breathless than normal he thought it could be the weather. Advised to increase salbutamol and to contact team if any further problems. Visit by clinician avoided” [Nurse 2]
Note	31	No action taken but relevant note input about the patient	“Nurse practitioner at GP practice informed that [patient’s] BP is usually low so will continue to observe” [Nurse 1]
Attempted contact	26	Tried to reach the patient but unable to do so	“Telephoned patient no answer message left to contact if any problems. Visit by Clinician Avoided” [Nurse 2]
Home visit	20	Date of a home visit or scheduled home visit in addition to those on the care pathway	“Patient visited today no complaints, given self-management advice and advised to contact if needed” [Nurse 2]
Hospital	7	Nursing notes indicated patient admitted to hospital	“Admitted to hospital [date] N1;” “Patient contacted by telephone and spoke with wife. She states that [patient] was admitted into hospital this morning following consultation with GP. Therefore Doc@home will not be completed through the next few days” [Nurse 3]

^a^ Orange “intervention” auto-generated by the system based on patient parameters. Nursing notes reflected the generation of orange level alert.

Alerts were categorized as “explained” and the rationale documented by nurses within the file, “conditional” as the alert was related to COPD, or an “error” in data input by the patient, as shown in Table 5. A review of patient metrics rarely occurred after a system-generated alert, showing that the clinicians were examining and monitoring patient parameters even when alerts were not being generated. Patient contact was almost always logged after an explained alert (n=61). Treatment interventions consisted of both lifestyle advice and medication adjustments. Only 20 home visit interventions took place during this pilot, and the nursing notes identified 27 instances in which a home visit was avoided through use of the telehealth-monitoring equipment.

**Table 5:** Clinical intervention subcategories, frequency, definitions, and examples (n=512).

Alerts	Total, n	Definition/inclusion criteria	Examples
Explained	61	Nursing notes indicated a reason for the alert	“Telephoned patient as alerted on being more tired than yesterday. Stated he felt he had just done too much on his allotment. Advised re: pacing, etc; no need to visit” [Nurse 2]
Conditional	447	Nursing notes reveal a connection to COPD causing the alert or unable to determine if the alert was explained so assumed to be triggered by the condition	“Telephone consultation [date]. Patient admits to not taking his inhalers as prescribed resulting n breathlessness. Advised to take his Atrovent and Salamol as instructed to improve condition” [Nurse not identified]
Error	4	Patient input error	“Telephoned patient who stated he had inputted without his oxygen on advised to input with oxygen on stated he was ok” [Nurse not identified]

Table 6 identifies the longest response times between a system-generated alert and a clinical intervention. Of the 260 interventions reported in the system, 152 were preceded by system-generated alerts. In all, 83 of these 152 interventions (54.6%) were delivered within 24 hours of the alert, with the remaining 69 of 152 interventions (45.4%) delivered more than a day after the alert was triggered.

**Table 6:** Top 10 longest response times following an alert.

Patient number	Intervention date (mm/dd/yy)	Intervention type	Preceding alerts (if any), n	Date of first preceding alert (mm/dd/yy)	Date of most recent preceding alert (mm/dd/yy)	Duration between first alert and intervention (days), n^a^
P14	07/15/11	Patient metric review	3	06/23/11	07/01/11	22
P15	07/15/11	Patient metric review	3	06/24/11	06/26/11	20
P9	03/18/11	Patient metric review	1	03/05/11		13
P16	07/12/11	Patient contact	3	07/03/11	07/11/11	9
P17	07/12/11	Patient contact	6	07/04/11	07/10/11	8
P6	03/11/11	Treatment	3	03/05/11	03/11/11	6
P16	07/01/11	Patient contact	6	06/26/11	06/30/11	5
P22	10/27/11	Patient contact	1	01/22/11		5
P13	06/01/11	Patient contact	6	06/27/11	07/01/11	4
P14	06/27/11	Patient metric review	1	07/23/11		4

^a^ Equals number of days between date of first preceding alert and intervention date.

If response times were greater than 1 week, a review of the preceding alerts identified that patients triggered a system alert for being more tired (n=2 alerts), requiring more relieving medication (n=6 alerts), or more tired and requiring more relieving medication (n=4 alerts). After generating system alerts for requiring more relieving medication on July 4 and 9, when patient 17 subsequently generated alerts for their blood pressure, sputum volume, and color on Sunday, July 10, contact was made with the patient within 48 hours.

Patient 6 generated system alerts for requiring more relieving medication than usual on March 5, 6, and 11. On March 4, the patient had been contacted by the service who requested that the patient’s general practitioner (GP) either visit in person or provide a prescription of antibiotics. The patient was completing the course of antibiotics in the 6 days that the alerts were generated. On Friday, March 11, the patient telephoned the service stating that they were “struggling with their breathing” and had completed their steroids and antibiotics. Although not at levels to trigger system alerts, the clinician noted that the patient’s physiological readings were deteriorating and recommended an out-of-hours GP visit, and arranged for a visit the following day by a community matron (experienced, community-based nurses who coordinate all the health and social care needs for patients with long-term or complicated health conditions).

### Health Service Usage

Eight of 23 patients were hospitalized for their COPD, three during the 8-week intervention and five during the 6 months following the intervention. In all, 14 patients (60%) triggered alerts but were able to remain at home. Of the 16 hospital admissions, five were for one patient. Only one patient had an A&E visit without being admitted. Five patients were taken to the hospital by ambulance and were admitted; their admission duration of zero days indicates that they were discharged that same day.

In this study, one patient (P3) was readmitted to hospital on day 5 of the intervention (their first day using the telehealth technology) and another (P13) on day 22 on the intervention after using the telehealth technology for 16 days. The third patient readmitted to hospital while on the intervention (P4) was readmitted on day 31 of the intervention.

All three patients who were hospitalized during the 8-week intervention had alerts prior to their hospitalization and received treatment interventions. One patient (P13) had alerts for 3 days consecutively prior to their hospitalization. On the first day, they generated alerts for two of eight NHS AECOPD key symptoms—being tired and having difficulty with self-care—in addition to alerts related to their blood pressure, SpO_2_ (%), and heart rate readings. On the second day, alerts were generated for their blood pressure, SpO_2_ (%), and heart rate readings. A clinician telephoned the patient and noted the patient said they “might not be inputting correctly.” The clinician noted that a visit was not required at this point, and that they would review the case again the following day to determine whether a home visit was necessary. On the third day, the patient again triggered alerts for their blood pressure, SpO_2_ (%), and heart rate readings, and the system generated an “orange” alert status for the patient. On the following day, before the service had an opportunity to arrange a home visit, the patient was admitted to the hospital and remained there for a week.

A second patient (P3) also generated system alerts prior to their hospitalization, which occurred on their first day of being on the intervention. They triggered an alert due to requiring more relieving medication. Later that day they were taken by ambulance to A&E and were subsequently admitted overnight. On discharge from the hospital, on their second day of using the system, they generated an alert because they were much more anxious than usual. On their third day, they again generated an alert due to their anxiety in addition to feeling more tired than usual and later that day their spouse contacted the service to say that the patient had again been admitted to hospital, although this admission was not recorded in the local hospital SUS data. This patient continued to require significant assistance with their condition and was admitted to hospital a further four times in the 6 months following discharge from the community telehealth-enabled service.

The third patient (P4) generated multiple system alerts almost every day they were on the intervention. The clinicians contacted the patient regularly and provided dietary advice to alleviate symptoms and recommended medication changes. In the week prior to their admission to hospital, this patient triggered multiple alerts every day except one (four days before their admission). The clinicians contacted the patient on four of the days alerts were generated. They also noted that the patient’s GP visited on the day before their hospital admission, and on the subsequent day when they recommended that the patient should go to the hospital. Once again, this admission was not recorded in the local hospital SUS data.

Seven of 39 (18%) treatment interventions administered to all patients throughout the study were provided to just one patient. Although this patient generated no more than one system alert per day, and always for the same prompt (“Have you had to use your relieving medication in the last 24 hours?”), there were ongoing medication adjustments and recommendations. The nursing notes also showed short periods of stasis after an alert in which the patient seemed to stabilize and reported feeling well. Despite the significant number of alerts, clinical assistance, and self-management advice, this patient was never hospitalized.

## Discussion

Our findings show that the volume, frequency, and type of alerts generated by a telehealth monitoring system is a good indicator of need for clinical contact and/or intervention for patients with early-stage COPD when first discharged from hospital following an exacerbation.

### Use of the Telehealth Monitoring System

Allowing for days in hospital, on holiday, or when there was a system fault when the patient could not reasonably be expected to use the system, patients failed to provide data on only 29 of 1175 days (2.47%) they had the system installed in their homes. Although little is currently understood around adherence to health technology interventions, or the required level of adherence for an effect on outcomes [21], this adherence level is higher than others seen in the telehealth field [22]. As a side note, holidays and hospitalizations were not taken into account in this project.

Only four alerts were triggered by patient input error. The ease of use of the equipment was later confirmed in interviews with the patients following completion of the intervention and discharge from the clinical service [23].

### Volume and Frequency of Alerts

As shown in Figure 1, the mean number of patients triggering alerts each day declined over the course of the intervention. This could imply that patients are more able to manage their disease, possibly as a result of the self-management advice provided and documented in the nursing notes, or that they experienced fewer symptoms.

It is currently unclear whether use of telehealth promotes or inhibits self-management behavior in people with COPD [24]. Telehealth can empower patients to self-manage their condition through facilitating increased knowledge of the condition and its symptoms [25], which supports the argument that participants in this study gained knowledge, became better at managing their condition, and triggered fewer alerts. However, it is also argued that telehealth may increase dependence on health care services, with reassurance provided through being “watched over” by health care professionals and the knowledge that they will intervene if something goes wrong [24]. To summarize, it is unclear why alerts reduced with time spent with the equipment, although it could be as simple as the fact that participants were recovering from a hospitalization when they first began using the equipment [20].

Although only 22 of 75 patient service days were delivered during December, one patient was responsible for all 28 alerts triggered that month. This patient generated a high number of alerts (n=77) throughout their time on the service, and was hospitalized twice while receiving the intervention, with one of these admissions occurring in mid-December.

### Alert Triggers

The most fundamental question upon which this entire analysis relies is whether an alert in the Doc@Home system means that the patient is experiencing an AECOPD. Although 12 system prompts and the four Bluetooth readings provided by the patients could trigger a system alert, there were three prompts—(1) “Have you had any problems in walking about in the past week?” (2) “Have you had problems doing your usual activities in the past week?” and (3) “Have you had to contact the following (health services) within the last 24 hours?”—that did not trigger system alerts regardless of the response entered by the patient. Similarly, answers to the question “How has your general health been in the last 24 hours?” were either not captured or were not reported in the data downloaded from the system. Because questions created on the telehealth system to be posed to participants are determined by clinicians, it would seem logical that all metrics should be set to trigger an alert if they indicate a significant decline in patient health status that would merit follow-up.

Two system prompts that did trigger system alerts—“Have you had to use your relieving medication in the last 24 hours?” and “How anxious have you been over the last 24 hours?”—did not appear to be tied to the NHS AECOPD list of key symptoms. Given that they both triggered a number of system prompts, we question whether they should be included in the key symptoms of an AECOPD.

There were no system prompts that could reasonably be linked to NHS AECOPDs symptoms of chest tightness or acute confusion. Although the latter may be difficult to incorporate into a remote monitoring system completed by a patient, the former could be easily included in the prompts and could alert clinicians to the onset of an AECOPD.

It is important to note that an explained alert does not mean that the patient’s COPD condition had no effect on triggering that alert. It means that, because of their COPD, as shown in Table 5, other variables—be they physical activity or the weather—exacerbated their condition, which then triggered a system alert. Simply having COPD was not the cause of the alert, but rather a confluence of factors. Jehn et al [26] explored the effects of heat stress (days warmer than 25°C) on AECOPDs, finding “heat stress negatively impacts clinical and functional status in patients with COPD and makes patients more vulnerable for disease-related morbidity.” In addition, the amount or type of activities of daily living undertaken may positively or negatively influence COPD symptoms, such as dyspnea and fatigue [27].

The care pathway indicated that patient-specific parameters for the four Bluetooth readings (blood pressure, heart rate, and SpO_2_) should be reviewed and amended following 10 days of data entry to eliminate unnecessary subsequent system alerts. Although it appears that some patients did have their parameters adjusted, this did not appear to occur for all patients. Patient 13 triggered system alerts for blood pressure, SpO_2_ (%), and heart rate for 17 of their 20 alerts.

### Clinical Response to Alerts

An AECOPD is a worsening of symptoms that, according to some definitions [12,15,28], can include a change in medication. There were 39 instances in which either a medication change or a lifestyle adjustment as a form of treatment was recommended to improve the patient’s health. Although the patient’s health status may have otherwise warranted a home visit, the telehealth monitoring system reduced unscheduled visits. This was confirmed in the nursing notes where the need for a visit had been averted in 26 of 360 clinician interventions; a search of the telehealth system data identified a “visit by clinician avoided” (n=4) and a “visit not required” (n=22). However, current evidence demonstrates that a reduction in health care utilization does not necessarily translate into cost effectiveness when looking at the overall cost of providing telehealth in comparison with usual care [29]. This study did not demonstrate cost savings for telehealth, and overall use of health care services increased [20].

The reason for the increased response time for patients who triggered more system alerts is unclear. The additional alerts may indicate a more complex patient requiring more time to develop a suitable care plan. Alternatively, the clinicians were acclimatized to the patients generating alerts more frequently. For example, for one patient the nursing notes state, “still struggles with ADLs [activities of daily living], but this is not new for [patient].” This particular patient generated the most alerts and experienced two hospitalizations during the course of their intervention and the 6-month follow-up period. Because difficulty with self-care is listed as one of the NHS AECOPD key symptoms, we question whether a more active response to recurring alerts may have halted the patient’s decrease in health status and ultimate hospitalizations.

A “treatment” was recommended only 11% of the time, with recommendations including referrals to pulmonary rehabilitation programs (PRPs). PRPs are designed to “help people with chronic lung problems” through “exercise and education...by a multidisciplinary team, which includes physiotherapist, respiratory nurse specialists, and dieticians” [30]. A search of the system data identified that the PRP option was only discussed with three patients on four separate occasions. We suggest that PRPs could be used more frequently as a treatment option to prevent or defer hospitalization. After days with treatment recommendations, such as days 10 and 11, the following days 12 to 15 showed a return to stability. When issues arose on day 18 through day 32, a variety of different treatment methods were administered by the nursing team and the patient’s GP. Subsequently, the patient’s health status stabilized and remained satisfactory for until day 52 when they were discharged from the service. We suspect that without this intervention, the options available to the patient would have been more limited or slower to access. The telehealth monitoring system seemed to help this patient overcome exacerbations of their COPD.

### Health Service Usage

As noted by Bentley et al [20], patients receiving the telehealth intervention were more than twice as likely to be admitted to hospital and for six times longer than the control group. This would seem to suggest, as in other studies [17,31,32], that telehealth monitoring fails to achieve the objective of keeping patients out of hospital or reduce health service usage. However, our analysis suggests that there may be a way to identify AECOPDs with enough time to intervene and thus prevent hospital readmission. Table 6 identifies an increase in alerts just before hospital admission for the three patients hospitalized during the intervention. Paired with the treatment intervention attempts, this suggests that the clinicians are recognizing the onset of an exacerbation and are attempting to intervene through treatment that includes medication changes, referrals to PRPs, and/or lifestyle advice.

The UK norm is 20% of patients are readmitted to hospital within 28 days of an AECOPD [30,34]. In this study, one of the three readmissions was on the first day of the intervention, so there was no opportunity for the service to have an impact. We are cautious of extrapolating from our relatively small sample size but, ignoring this admission, the hospital readmission rate of just 9% (2/23) suggests that the ability of this service to reduce the frequency of hospital readmissions merits further investigation. Although two of the patients saw an increase in alerts for the captured metrics before hospitalization, the third patient did not, so it is not possible to say that the metrics used can definitely predict an AECOPD. Two patients were recommended to go to hospital by their GPs, suggesting that the nursing team and/or patient engaged other health care providers in an effort to exhaust other options before returning to hospital. It appears as though increased effort, clinician attention, medication, and system funding were expended on these patients and yet a readmission was not avoided. The service was designed to support early intervention, which is critical for preventing worsening of an AECOPD [33], but NHS restructuring, the loss of a key champion for the trial staff, and attrition resulted in an eventual total loss of 60% of staff capacity within the frontline clinical team. With this significant decrease in resources, the clinicians clearly used the system to identify patients who demonstrated greatest need of support, and did their utmost to contact these patients and to alleviate their symptoms.

### Interrogation of System Data

Although telehealth monitoring systems are designed for analysis of data by clinicians at the time of care delivery, retrospectively downloaded data has proved to be highly time consuming and complex to analyze. With only 23 participants in this study, we were able to undertake this task; for a larger study, this could prove to be insurmountable unless the data could be downloaded in a format better suited to analysis. We question whether this is the reason system data analysis has not been undertaken or published to date. These data provide critical insights into service provision and patient profiles; therefore, technology providers need to consider not only frontline care providers, but also staff conducting clinical audits, service improvement studies, or research. The uptake of a new intervention requires analysis of all evidence available to demonstrate effectiveness. It seems rather ironic that an intervention designed to capture and report remotely provided data is unable to better support retrospective analysis, and we strongly encourage technology providers to ensure that this current deficiency is addressed.

### Study Limitations

The SUS information taken from the PCT NHS records showed some discrepancies when compared to the telehealth system nursing notes that would have affected analysis if they had not been identified. For example, a hospital admission was not shown in the SUS data; however, the telehealth system nursing notes included specific details about a hospital admission of at least 3-days duration. It is possible that the patient was hospitalized outside of the PCT for the region in which the study was conducted and consequently would not be captured within their SUS dataset.

When the clinician viewed the telehealth system at the time of care delivery, the display included color-coded alerts (red, orange, or yellow) [15]. When the same data are retrospectively downloaded from the server for research purposes, with the exception of the orange alert (see Table 4), these color codes were not identified. Alerts were only identified as dichotomous “yes” or “no” nominal data. Consequently, there is no way to determine what specific parameters caused alerts to be yellow, orange, or red. It is also not possible to tell whether an alert had previously been displayed at a yellow level and a change to orange indicated an increase in priority or conversely if the previous alert was red and a change to orange indicated a decrease in urgency. Consequently, our analysis has been restricted to the dichotomous data available to us.

### Conclusions

The pilot RCT did not identify a reduction in health care usage; in fact, it had a higher rate of usage among the telehealth group relative to the control [20]. However, from our system data analysis we suggest that this telehealth monitoring-supported service could fulfill an important role: enabling patients with COPD to better manage their condition. Although we question whether the addition of further prompts to assess all the key NHS AECOPD symptoms would further improve the system, the prompts used during this study did seem able to identify when a patient may be experiencing an exacerbation of their COPD and may require clinical intervention. Identification of an AECOPD is insufficient and, to alleviate symptoms and enable a patient to remain at home, timely intervention is required. Where service resourcing limits service capacity, a delay in responding to system-generated alerts can result in patients being hospitalized, thereby negating the value of the intervention and the service. It is clear—predominantly through remote interaction with the patient—that during the intervention, the service successfully enabled a number of patients to remain at home despite exacerbations in their COPD.

Some of the patients were clearly unstable following their discharge from hospital, triggering a high number of system alerts. With a larger study to eliminate the impact of a small number of more complex patients, and more consistent clinical resourcing [20] to enable timely intervention, we question whether the outcomes of the pilot study would have been somewhat different.

We recommend running a larger study using telehealth monitoring with prompts to assess all 10 of the NHS AECOPD symptoms in addition to the two non-NHS AECOPD-related prompts that generated alerts in this study to determine which prompts may assist in the accurate identification of an AECOPD. To test whether removal of the system does defer hospitalizations, we further suggest conducting the study such that following a standard “intervention” period, in which one study group has the service removed, while another continues to use the service for a comparable 6-month period to determine whether there is any difference between group hospitalization rates.

## Abbreviations

A&E: Accident and Emergency
AECOPD: acute exacerbation of chronic obstructive pulmonary disease
COPD: chronic obstructive pulmonary disease
GP: general practitioner
NHS: National Health Service
PCT: Primary Care Trust
PRP: pulmonary rehabilitation program
RCT: randomized controlled trial
SUS: Secondary Uses Service
